# iPSCs are safe!

**DOI:** 10.1186/s13578-017-0157-3

**Published:** 2017-05-30

**Authors:** Hualong Yan, Yun-Bo Shi, Jing Huang

**Affiliations:** 1 Cancer and Stem Cell Epigenetics, National Cancer Institute, National Institutes of Health, Bethesda, MD USA; 2 Molecular Morphogenesis, National Institute of Child Health and Human Development, National Institutes of Health, Bethesda, MD USA

**Keywords:** Induced pluripotent stem cells (iPSCs), Cloning, Cell therapy, Genomic alterations, Reprogramming

## Abstract

Induced pluripotent stem cells (iPSCs) hold great promises in cell therapy. However, the potential safety issues have dampened the enthusiasm of their clinical development. One of the biggest concerns came from the observations that genomic alterations exist in iPSCs. Using next generation sequencing of clonal skin fibroblasts and the iPSC clones derived from the same skin fibroblasts, Dr. Liu and his colleagues in the National Human Genome Research Institute, National Institutes of Health (NIH), USA, in collaboration with Dr. Dunbar’s group in the National Heart, Lung, and Blood Institute, NIH, USA, have now elegantly demonstrated that most of the observed genomic alterations in iPSCs were inherited rare alterations from the parental cells. Their findings suggest that reprogramming process does not appear to be more mutagenic than simple subcloning of cultured cells and that iPSCs are safe for cell therapy.

Ever since the breakthrough development of the induced pluripotent stem cells (iPSCs) technology a decade ago, iPSCs have attracted immense attention for cell therapy toward human diseases. Before iPSCs can be used in cell therapy, it is critical to know whether the reprogramming process leads to significant mutations or structural variations in the genome that may be detrimental to human health. Several early studies revealed the existence of genomic alterations in iPSCs, including single nucleotide variations (SNVs), copy number variations (CNVs), and chromosomal rearrangements [1–5]. There are two possibilities for the observed genomic alterations in iPSCs. One is that reprogramming stress and/or long-term in vitro culture select alterations favoring the reprogramming process [1–3]. The second is that iPSCs inherit rare alterations from the parental cells, which are heterogeneous in terms of genomic alterations [1, 4, 5]. These early studies used whole genome or exome sequencing of clonal iPSCs and pooled parental source cells and had only a limited sequencing depth that allowed the detection of only genomic alterations/variations with a frequency higher than 0.01 in parental cells. Thus, it remains possible that many of the genomic alterations were inherited from rarer alterations from the parental cells.

Now, a team of researchers led by Dr. Liu of the National Human Genome Research Institute, National Institutes of Health, USA, has used a novel approach to address this critical question. Dr. Liu and his colleagues derived clonal skin fibroblasts and iPSCs and performed whole-genome exome sequencing and single nucleotide polymorphism analyses [6]. They identified hundreds of SNVs, CNVs and chromosomal rearrangements compared to pooled parental skin fibroblast cells. Importantly, the clonal skin fibroblasts and clonal iPSCs have similar number of genomic alterations. This demonstrates that compared to simple subcloning, the reprogramming process does not generate more mutations, which potentially could make iPSCs or their derived cells more tumorigenic. They have further carried out targeted sequencing on the regions of these hundreds of genomic alterations with greatly enhanced sequencing depth that allowed them to detect genomic alterations with a frequency of 0.0001 in the parental skin fibroblasts. Using this trick, they discovered that most observed alterations existed in the parental skin fibroblasts in low frequencies, indicating that iPSC reprogramming is not mutagenic.

It is also worth noting that some of the iPSCs in this study were generated by using episomal vectors expressing several factors including Oct4, Nanog, Sox2. cMyc was replaced by L-Myc and p53 was transiently suppressed by shRNA [7]. Given that p53 is such a potent tumor suppressor and its loss tends to be associated with genome instability, it is interesting that this reprogramming regime did not generate more mutations. This suggests that the transient loss of p53 does not affect the cell’s ability to maintain genome integrity but enhances its capacity to be reprogrammed.

In summary, the findings from Dr. Liu and his colleagues provide convincing evidence for the lack of mutagenic effects by iPSC reprogramming stress and that iPSCs should be safe for use in cell therapy.
